# Nuclear Magnetic Resonance Based Metabolomics—A Rising Star in Traditional Chinese Medicine Research

**DOI:** 10.3390/ph18081186

**Published:** 2025-08-12

**Authors:** Yu Hu, Lei Liu, Guangli Yan, Luoning Bai, Le Yang, Ling Kong, Hui Sun, Chang Liu, Ye Sun, Ying Han, Xijun Wang

**Affiliations:** 1 State Key Laboratory of Dampness Syndrome, The Second Affiliated Hospital Guangzhou University of Chinese Medicine, Dade Road 111, Guangzhou 510120, China; 18845639230@163.com (Y.H.); leyang92gzy@sina.com (L.Y.); sunye0126@126.com (Y.S.); 2 State Key Laboratory of Integration and Innovation of Classic Formula and Modern Chinese Medicine, National Chinmedomics Research Center, National TCM Key Laboratory of Serum Pharmacochemistry, Metabolomics Laboratory, Department of Pharmaceutical Analysis, Heilongjiang University of Chinese Medicine, Heping Road 24, Harbin 150040, China; liulei7711@126.com (L.L.); gancaosuan@163.com (G.Y.); bailuoning2024@163.com (L.B.); 15244624557@163.com (L.K.); lc_work_lc@163.com (C.L.); hanying314@sina.com (Y.H.)

**Keywords:** efficacy, metabolomics, nuclear magnetic resonance, traditional Chinese medicine

## Abstract

Metabolomics, a promising field in the realm of omics, focuses on the investigation of alterations and patterns in the composition and abundance of metabolites generated by organisms under perturbation, directly linking measurable chemical reactions with biological events. Its research philosophy aligns harmoniously with the holistic perspective and syndrome differentiation and treatment principles of traditional Chinese medicine (TCM). Consequently, metabolomics has garnered unparalleled attention and has been widely applied in various fields of TCM research such as disease diagnosis, effective constituents and mechanism related with efficacy. In recent years, nuclear magnetic resonance (NMR)-based metabolomics, a non-destructive testing technique, has played a crucial role in metabolomics research, owing to its exceptional repeatability, stability, and advantages in qualitative and quantitative aspects. Through reviewing relevant literature in recent years, this article provides a comprehensive analysis of the fundamental principles of NMR metabolomics technology and its utilization in TCM. Additionally, it examines the challenges encountered in this field and explores potential future development trends, aiming to offer substantial support for further investigations in the realm of TCM metabolomics.

## 1. Introduction

For thousands of years, traditional Chinese medicine (TCM) has accumulated valuable experience in clinical application. Based on the unique multi-component and multi-target characteristics, it holds an irreplaceable position in the treatment of complex and chronic diseases [1,2,3]. Especially when coronavirus disease 2019 (COVID-19) rapidly swept the world, the important role played by TCM has given the world a new understanding of it [4,5,6]. However, a unique theoretical system is difficult to define scientifically, and the effective constituents and mechanism of efficacy are not clear, which are key factors restricting the modernization and internationalization of TCM. Therefore, establishing a bridge between TCM and modern science has become the main focus of current medical research [7,8].

Metabolomics, one of the important subdisciplines of systems biology, reflects a series of biological events that occur in a certain pathological or physiological process by revealing the overall changes in metabolism under the influence of internal and external factors, and its emergence can be traced back to the 1990s [9]. As the most downstream “omics” in systems biology, metabolomics mainly uses high-throughput methods to qualitatively and quantitatively analyze metabolites in cells, tissues, or organisms, thereby directly linking measurable chemical reactions with biological events. And it is the most closely related “omics” to biological phenotypes [10,11,12]. The research philosophy alignment is highly compatible with the holistic perspective and syndrome differentiation and treatment principles of the TCM theory system. Therefore, it has been widely applied in the study of scientific issues such as the effective constituents and mechanism of TCM efficacy, and the compatibility effects of prescriptions [13,14,15]. In recent years, Professor Wang has organically integrated metabolomics with TCM and proposed the research method of “Chinmedomics”, providing new ideas and approaches for the modernization of TCM [16,17,18].

So far, mass spectrometry (MS) and nuclear magnetic resonance (NMR) spectroscopy are the most powerful approaches in metabolomics research [19,20]. Although MS has high sensitivity, the detection results of metabolites are greatly influenced by ionization conditions and specific instrument selection. In addition, complex sample handling, reliance on standards and databases, and weak identification of isomers all limit the deeper application of MS in metabolomics [21,22]. Compared to MS, NMR has many unique advantages: simple sample handling, wide dynamic range, high-throughput and non-destructive data acquisition, rich spectral information, etc., all of which can improve the accuracy of metabolite identification. Additionally, NMR can not only achieve absolute qualitative and quantitative analysis of different types of metabolites simultaneously, but also has unparalleled repeatability and stability. This means that researchers can accumulate data from different laboratories or at different times, achieving true information sharing. Most importantly, NMR-based metabolomics allows the identification of completely unknown metabolites, including an accurate distinction of isomers [23,24].

**Figure 1:**
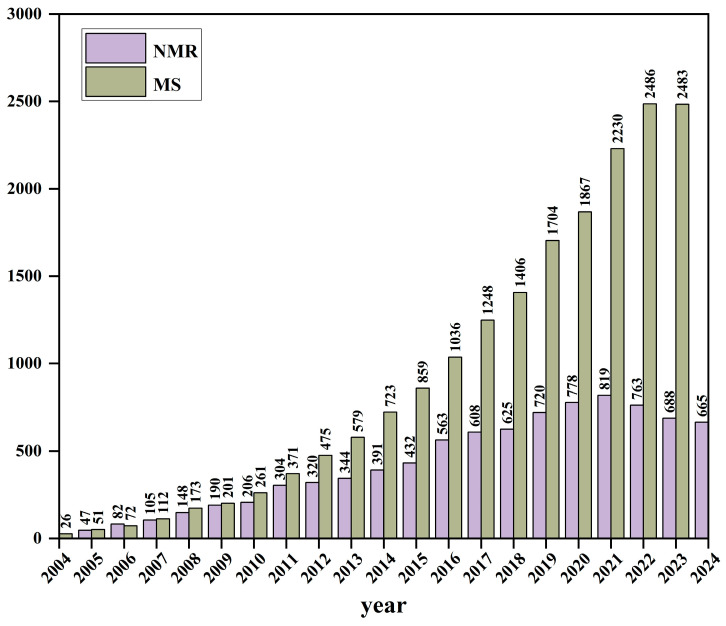
Annual trends in publications (2004–2024) of metabolomics studies utilizing MS (yellow) versus NMR (purple). Data were obtained from Web of Science using the search terms “metabolomics and MS” and “metabolomics and NMR”, respectively.

However, past metabolomics studies have predominantly relied on MS, with NMR receiving far less attention. Although interest in NMR-based metabolomics has grown in recent years, its potential remains underexplored (Figure 1). This review highlights representative applications of NMR in metabolomics, discusses current challenges, and outlines future trends, aiming to draw more attention to this promising field and provide new insights for the modernization of TCM.

## 2. Introduction to NMR

### 2.1. Basic Principles

**Figure 2:**
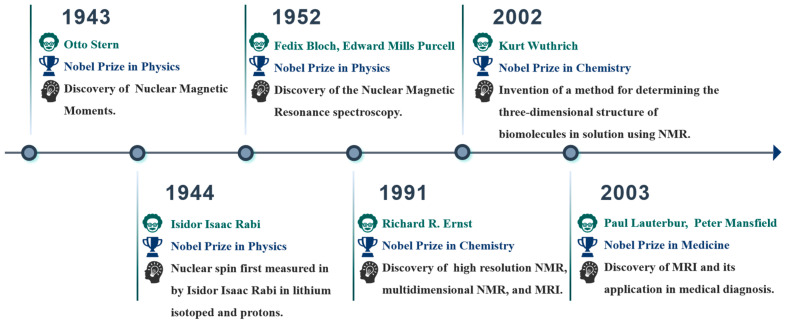
Nobel laureates who have made outstanding contributions to the development of NMR.

NMR has undergone remarkably rapid development (Figure 2), evolving from Pauli’s nuclear spin theory in 1924 to the independent discovery and confirmation of NMR phenomena by Edward Mills Purcell and Felix Bloch in 1945, and subsequently to the development of Fourier transform technology [25,26,27,28]. Since the 1970s, advanced NMR techniques, including solid-state NMR, two-dimensional NMR, magic-angle spinning (MAS), and Magnetic resonance imaging (MRI), have emerged. Within just a few decades, NMR has become an indispensable research tool across multiple disciplines such as chemistry, food science, medicine, biology, genetics, and materials science [29,30,31,32,33].

**Figure 3:**
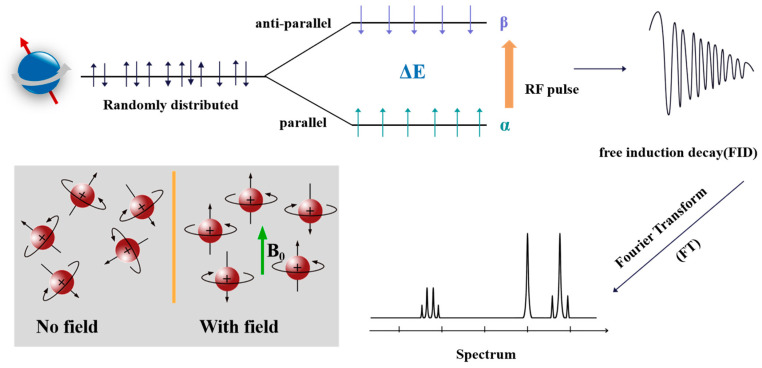
The schematic diagram of the basic principle of NMR signal generation.

NMR, as the name suggests, consists of three parts: “Nucleus”, “Magnetic field”, and “Resonance perturbation”. As is well known, nuclei are composed of protons and neutrons, both of which undergo spin. The combination of the two determines the overall spin of the nucleus. Simply put, nuclei with non-zero spin absorb appropriate frequency electromagnetic waves in magnetic fields and undergo energy level transitions, resulting in NMR signals (Figure 3). It should be noted that only when the frequency of the radio frequency (RF) field is the same as the frequency of nuclear spin precession, can it assist with energy level transitions [34].

Different nuclei have different spins. Specifically, the nuclei of ^16^O, ^12^C, ^32^S, ^28^Si, etc., are all characterized by spin zero, so there is no magnetic moment, which makes them unable to be studied using NMR [35].

Nuclei with spin 1 or greater can be compared to an ellipsoid, with uneven charge distribution and non-zero electric quadrupole moment, such as the nuclei of ^11^B, ^35^Cl, ^79^Br, ^81^Br, ^2^H, ^14^N, and ^17^O, etc. Therefore, they have a special relaxation mechanism that often leads to broadening of NMR signals, which is unfavorable for NMR studies [35].

In comparison, the nuclei with spin 1/2 can be regarded as a sphere with uniformly distributed nuclear charges and spins like a gyroscope, resulting in the formation of magnetic moments. They are the main objects of NMR research, such as ^1^H, ^19^F, ^31^P, ^3^H, and ^13^C. The abundance of the first three in nature is close to 100%, which makes them easy to measure [35]. However, as the main constituent element of organic compounds, ^12^C is not suitable for NMR detection, and the abundance of ^13^C is very low (1.1% of total carbon). Moreover, the gyromagnetic ratio of ^13^C is only a quarter of ^1^H, so the sensitivity of ^13^C-NMR is much lower than that of ^1^H-NMR [24]. Therefore, it is not difficult to understand why scholars in current popular metabolomics research focus more on the ^1^H rather than other nuclei [36,37,38], not only because it is included in the vast majority of metabolites, but also because of its sensitivity. Notably, although most metabolites do not contain P atoms and the sensitivity of ^31^P is lower than ^1^H, the natural abundance of ^31^P is high. Therefore, in metabolomics research, ^31^P-NMR primarily focuses on the study of key phosphorus-containing compounds involved in energy metabolism, such as phospholipids and nucleoside metabolites (ATP, GTP, NADP, etc.) [39,40,41].

### 2.2. Characteristics of NMR

**Figure 4:**
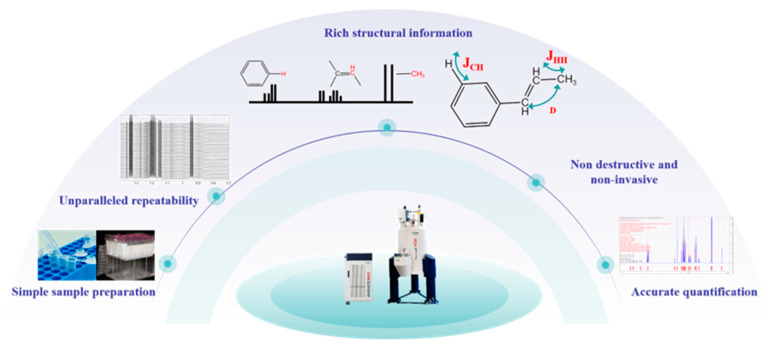
The dazzling characteristics of NMR analysis technology.

The most extraordinary feature of NMR is its non-destructive and non-invasive detection (Figure 4), whether for living organisms, intact tissue, or other samples in solid, liquid, or gaseous form [34,42,43,44], which is determined by its principles. As mentioned previously, the core of NMR technology is the interaction between the spin properties of atomic nuclei and external magnetic fields, which does not involve chemical or physical changes to the sample. This characteristic of NMR makes it particularly suitable for the analysis of complex biological samples, such as plasma/serum and urine [45]. Notably, high resolution magic-angle spinning (HR-MAS) NMR is particularly valuable for analyzing semi-solid samples such as biological tissues and cell suspensions, as it preserves in situ metabolic information while allowing sample recovery for subsequent analyses [46,47,48]. This unique capability enables comprehensive multimodal correlation analysis of the same specimen to comprehensively elucidate molecular mechanisms while maximizing the utility of precious biological materials.

Compared to other tools, NMR can provide redundant spectral information, such as chemical shift, integral area, J-coupling, dipolar coupling, relaxation, and Overhauser effect (NOE). So, the assignment of the vast majority of known or unknown metabolites, natural products, and nucleic acids can be easily accomplished, and even the discrimination of isomeric compounds that are particularly challenging to resolve by LC-MS has transitioned from a theoretical possibility to a practical reality [49,50,51].

The unparalleled reproducibility and stability are also significant advantages that must be highlighted [52]. It not only minimizes experimental variability, but also allows the integration of datasets acquired from different instruments, multiple sites, and various times. This is of paramount importance for discovering biomarkers and advancing laboratory research findings into clinical practice.

Over a decade ago, studies demonstrated that NMR could achieve precise quantification across an exceptionally wide dynamic range (>6 orders of magnitude) with errors within 2%, without the need for additional concentration standards [53]. This was accomplished by implementing sufficient relaxation delays to avoid signal saturation, using small pulse angle excitations to prevent radiation damping, and maintaining fixed receiver gain with rigorous RF homogeneity control. Notably, narrow linewidths and hardware consistency are essential prerequisites for maintaining measurement precision. Nowadays, the unique quantitative advantages make NMR irreplaceable in fields such as chemistry, materials science, and environmental monitoring, especially in the pharmaceutical industry. As you can see, researchers can accurately identify and quantify biomarkers, which is of great significance for disease diagnosis, prognosis evaluation, and treatment efficacy monitoring. Furthermore, by analyzing the changes in specific metabolites in the patient, the most suitable treatment plan can be further developed to achieve personalized treatment, which is also a hot topic in current research [54].

However, low sensitivity is an inherent drawback of NMR. Both enhancing magnetic field strength and optimizing probe design represent effective approaches to enhance sensitivity. Despite significant progress, the battle over sensitivity is still ongoing [55].

## 3. NMR-Based Metabolomics

Metabolomics, a new discipline that emerged in the 1990s, studies the dynamic changes of endogenous metabolites (<1500 Da) in organisms to elucidate the mechanisms underlying pathological and physiological processes [56]. Its research philosophy is consistent with the holistic and dialectical views of TCM, and its application provides a new approach for the modernization of TCM [57].

**Figure 5:**
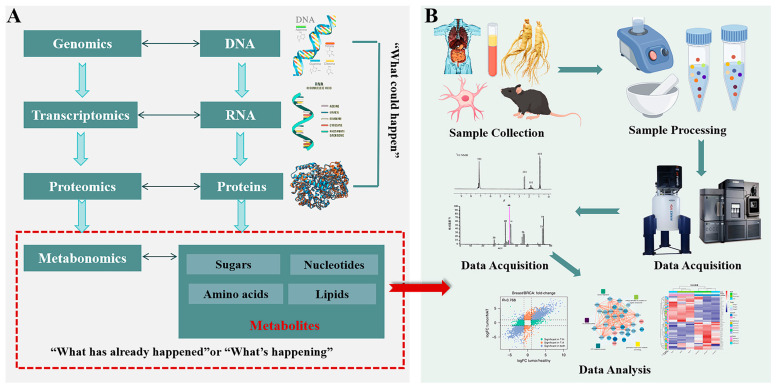
A brief description of metabolomics. (**A**) The relationship between genomics, transcriptomics, proteomics, and metabolomics. (**B**) The general process of metabolomics studies.

As the most downstream omics in systems biology, metabolomics collaborates with genomics, transcriptomics, and proteomics [58,59,60,61]. To put it bluntly, it can amplify subtle changes occurring at the genetic and protein levels, facilitating detection while directly reflecting ongoing biological events [62] (Figure 5). According to different research objectives, metabolomics can be divided into untargeted and targeted studies. Just as the term suggests, the former is an unbiased analysis that aims to comprehensively detect as many metabolites as possible, commonly used for the discovery of biomarkers. The latter is a targeted detection method mainly used to verify the results of untargeted research or as a validation tool for biological pathways. The research process of metabolomics generally includes sample collection and pretreatment, data acquisition and processing, and biological interpretation [63,64,65], and the common analytical tools are MS and NMR [66]. The continuous increase in related publications indicates the rapid development of metabolomics since its inception. However, there are still many challenges in the coverage and accuracy of qualitative and quantitative detection due to the diverse types, complex structures, and significant differences in the content of metabolites [24].

With this, it is clear that NMR is highly favored in metabolomics research due to its non-destructive, flexible, highly reproducible, and precise characteristics. In the following paragraphs, how NMR is applied to metabolomics research will be presented from the perspective of its general research steps.

### 3.1. Sample Preparation

Undeniably, reasonable sample collection and processing are the primary steps to ensure ideal and reliable results [67]. Biological samples used for metabolomics studies are inherently complex. When MS, a technique that was widely used in the past, is employed, complex preprocessing on the samples, such as protein precipitation, which would introduce risks and increase the complexity of analysis, is often required. In contrast, in NMR-based metabolomics research, certain techniques can reduce the need for pretreatment. For example, the CPMG (Carr-Purcell-Meiboom-Gill) pulse sequence can suppress protein signals, and the pre-saturation pulse sequence can attenuate water signals. These methods are not only more time-efficient, but also prevent the introduction of experimental variation. In fact, NMR-based metabolomics only requires minimal sample processing before spectral acquisition and allows the collection of the whole metabolic fingerprint [45].

Any biological fluid can be used for NMR-based metabolomics research, but in practice, blood and urine are the most commonly used due to their ease of accessibility. The European Consensus Expert Group Report has designated the general recommendations that biobanks should follow [68]. Furthermore, researchers have used NMR to evaluate the effects of different pre-treatments on the quality of urine and blood samples, and proposed standard operating procedures (SOPs) for sample pre-treatment in metabolomics studies to standardize certain variables, such as time delay before sample preparation or centrifugation methods [69]. It should be noted that blood is usually tested in the form of serum or plasma, rather than being used directly. This is because hemoglobin in blood is paramagnetic, which not only causes inhomogeneity of the local magnetic field, resulting in spectral broadening and reducing resolution and signal-to-noise ratio (S/N), but also induces displacement in chemical shift and reduction in relaxation time. The disappearance or indistinguishability of some signals is an inevitable result [35,70]. However, a serious drawback of plasma/serum metabolomics is its weak ability to measure and evaluate crucial metabolites related to redox and energy metabolism, such as NAD, NADH, ATP, ADP, GSH, and GSSG. Recently, Nagana Gowda and Raftery have developed a ^1^H-NMR metabolomics approach using whole blood, which enables the detection of these unstable species present at high concentrations in red blood cells without affecting other metabolites. This study is expected to open a new chapter in blood metabolomics [71].

For cell and tissue samples, which are also commonly used in NMR-based metabolomics, there are two distinct approaches to choose from: one is to directly use the intact tissue or cells without any pretreatment, while the other involves extracting metabolites prior to analysis [72,73]. The former approach preserves the metabolites in their native state, providing a more authentic reflection of complex biological conditions. It is applicable for capturing the metabolic dynamics of intact cells or tissues and understanding metabolic networks and intracellular interactions. This is very appealing, but just like a coin has two sides, it comes at the cost of complex signals, which means that higher resolution or multiple experiments are required to identify the metabolites. In fact, the latter is more commonly used. By employing appropriate extraction methods, it is possible to remove the majority of non-target substances and impurities, thereby simplifying the spectra and making target signals clearer, and it is particularly suitable for the detection of low-abundance metabolites. Of course, a range of extraction methods is feasible, with the perchloric acid method being the most commonly used for extracting water-soluble metabolites. In addition, chloroform/methanol extraction, acetonitrile/water extraction, and methanol/ethanol/water extraction are also alternative options, which partition metabolites into polar metabolites, non-polar metabolites, and protein particles [74,75].

Before NMR analysis, the sample is typically dissolved in deuterium oxide (D_2_O) [76]. On the one hand, this is to minimize solvent interference with the metabolite signals and improve S/N. On the other hand, it facilitates real-time monitoring and correction of the magnetic field, a process commonly referred to as “lock”. Of course, other solvents such as deuterated chloroform (CDCl_3_), deuterated methanol (CD_3_OD), deuterated dimethyl sulfoxide (DMSO), and deuterated acetone (C_3_D_6_O) are also available [77,78]. Choosing the appropriate deuterated solvent is crucial, and the specific choice primarily depends on the polarity of the target metabolites. Simply put, CD_3_OD is suitable for water-soluble metabolites, CDCl_3_ is ideal for lipid-soluble metabolites, and C_3_D_6_O works well for moderately polar metabolites. For some special metabolites that are insoluble in other solvents, DMSO is often a good choice. In addition, adding an appropriate internal standard is key to calibrating chemical shifts and improving the accuracy and reliability of results, and its selection is also related to the polarity of the target metabolite. Typically, [2,2,3,3-_2_H_4_]3-(trimethylsilyl)propionic acid sodium salt (TSP-Na) and 2,2-dimethyl-2-silapentane-5-sulfonic acid sodium salt (DSS-d_6_) are used for the analysis of water-soluble metabolites, with DSS being more stable under acidic conditions, while tetramethylsilane (TMS) is used for the detection of non-polar metabolites [79,80,81,82]. Last but not least, due to the influence of pH, the chemical shifts of certain resonances vary from sample to sample, which poses challenges for the identification of metabolites. Therefore, phosphate buffer solution needs to be added during the sample preparation process to stabilize the pH [69]. Finally, the prepared sample was transferred into high-resolution 5-mm or 3-mm NMR tubes for NMR spectroscopic analysis.

Here are two representative protocols of sample preparation for NMR metabolomics (one from tissue extracts and the other from serum) [83]: “Esophageal tissue slices were homogenized in a solution containing distilled water and methanol. Then, chloroform and distilled water were added. The supernatant was collected and evaporated and then redissolved in 550 μL of PBS/D_2_O buffer (0.1 M, pH 7.4) and 50 μL of TSP/D_2_O stock solution. 500 μL of the supernatant was transferred into a 5 mm NMR tube for ^1^H NMR spectroscopic analysis. The second extraction protocol requires to add 400 μL of each serum sample to 200 μL of PBS/D_2_O buffer, and the mixtures were vortex-mixed for 60 s. After centrifuging the mixture at 10,000 rpm for 5 min, 500 μL of the supernatant was used for ^1^H NMR spectra acquisition.”

### 3.2. Data Acquisition

**Figure 6:**
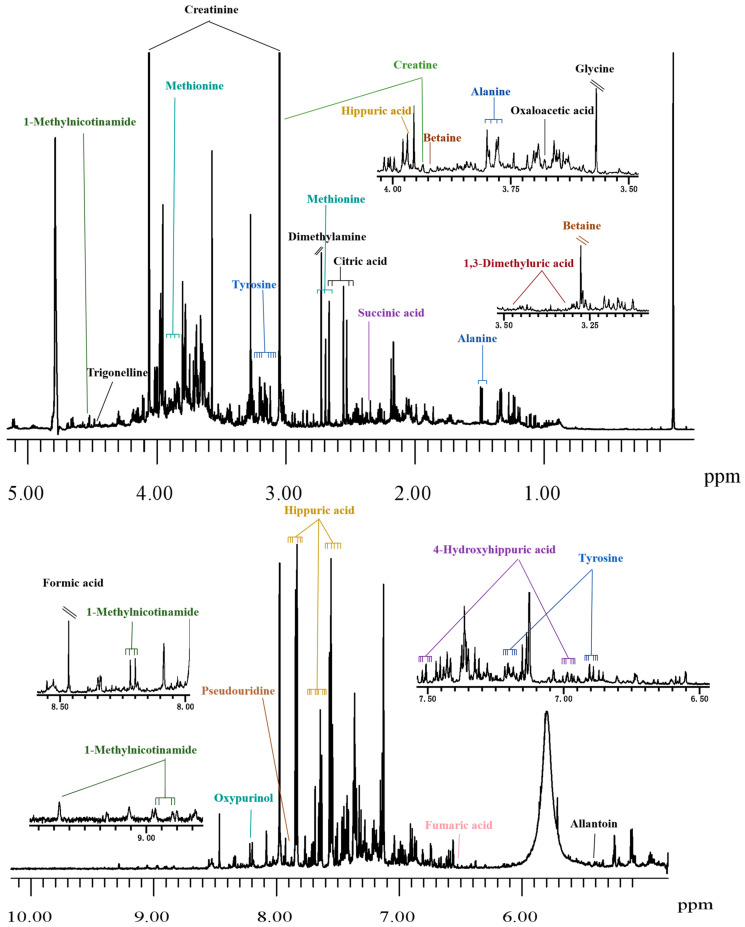
A typical 600 MHz 1D CPMG ^1^H-NMR spectrum of a urine sample obtained from healthy individuals in our laboratory. The urine supernatant was mixed with buffer (1.5 M KH_2_PO_4_, 1 mM TSP (sodium trimethylsilyl propionate-[2,2,3,3-_2_H_4_]), 0.13 mM NaN_3_ in D_2_O, pH 7.4 ± 0.1) and measured at 300 K (32 scans, 65,536 data points, spectral width of 11,904.76 Hz).

**Figure 7:**
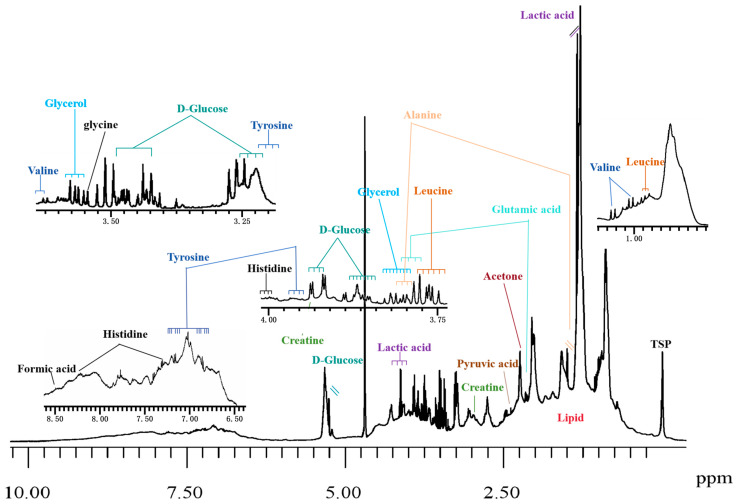
A representative 600 MHz 1D NOESY ^1^H-NMR spectrum of a serum sample collected from healthy individuals in our laboratory. The serum sample was mixed with buffer (75 mM Na_2_HPO_4_, 2 mM NaN_3_, 4.6 mM sodium trimethylsilyl propionate-[2,2,3,3-_2_H_4_] (TSP) in H_2_O/D_2_O 4:1, pH 7.4 ± 0.1) and measured at 310 K (32 scans, 98,304 data points, spectral width of 17,857.14 Hz).

So far, one-dimensional (1D) ^1^H-NMR-based metabolomics is most commonly used, exhibiting very narrow line-widths (typically less than 1 Hz) and thus possessing exceptional resolution. Generally speaking, a representative ^1^H-NMR spectrum of urine contains thousands of signals, which are primarily attributed to small molecular metabolites such as amino acids, ketone bodies, lactate, acetate, etc. (Figure 6). Due to low molecular weight and rapid diffusion, these metabolites exhibit sharp lines. In contrast, the spectra of plasma/serum are more complex, encompassing both low- and high-molecular-weight components (Figure 7). Proteins and lipoproteins typically appear in low field regions and provide broad bands, while the sharp signals of low molecular weight metabolites are either superimposed on or overlapped with the broad bands of proteins and lipoproteins [73].

Interference signals can be effectively eliminated through the adoption of suitable experimental methods. In most NMR-based metabolomics studies, 1D nuclear Overhauser enhancement spectroscopy (NOESY) is preferred for water suppression, and CPMG as a T2 filter to remove macromolecule signal [84,85]. They are essentially complementary. Nonetheless, it is undeniable that certain deficiencies persist. Reassuringly, not only are there many established alternative pulse sequences, such as ES and WATERGATE [86], which use gradient pulses and selective excitation techniques to suppress specific signals, as well as WET [87] and PURGE [88], which suppress water peaks through T1 relaxation differences and a combination of presaturation pulses and gradient echo techniques, respectively, but researchers are also continuously developing new ones [89].

**Figure 8:**
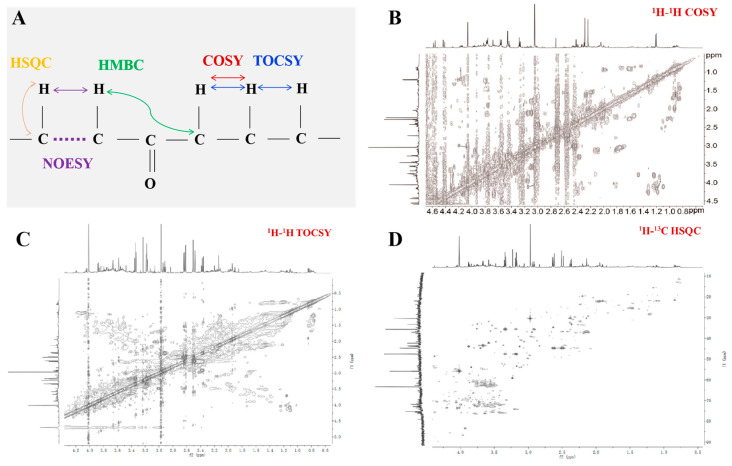
Common 2D NMR experiments. (**A**) What information can we obtain from these experiments?; (**B**) ^1^H-^1^H COSY spectrum of urine sample (adapted from [95]). The sample was prepared by mixing 400 µL urine with 200 µL phosphate buffer (0.2 M, Na_2_HPO_4_/NaH_2_PO_4_, pH 7.4) and 100 µL D_2_O (1% TSP). The spectra were obtained at 298 K on a 500 MHz spectrometer (Bruker, Hamburg, Germany); (**C**,**D**) ^1^H-^1^H TOCSY (**C**) and ^1^H-^13^C HSQC (**D**) spectrum of urine sample (adapted from [96]). The sample was prepared by mixing 400 µL urine with 200 µL 1.5 M KH_2_PO_4_ buffer (pH 7.4, 0.05% NaN_3_, 100% D_2_O, and 0.1% TSP). Spectra were acquired at 300 K on a 500 MHz NMR spectrometer system (Varian, Palo Alto, CA, USA).

Severe signal overlap is a tricky issue faced by 1D NMR, while 2D NMR technology disperses signals into two dimensions (such as ^1^H-^1^H, ^1^H-^13^C), which can partially alleviate this problem and provide additional resolution, enabling the detection and identification of more metabolites. As a result, it has gained favor among researchers and is increasingly applied in metabolomics studies. What is even more commendable is that statistically relevant changes in low-abundant metabolites can be better characterized using 2D NMR. Although they are more complex and time-consuming compared to 1D NMR, with the advancement of rapid sampling techniques (such as non-uniform sampling [90]), they are fully capable of high-throughput metabolomics research. 1H-1H correlation spectroscopy (COSY) is the simplest, yet most widely used among all 2D NMR experiments, providing coupling relationships between protons and helping to identify adjacent protons. ^1^H-^1^H total correlation spectroscopy (TOCSY) is an extension of the COSY that provides long-range coupling relationships between protons, aiding in the identification of all protons within the same spin system, such as the signals from individual sugar rings in polysaccharides or individual amino acid residues in proteins. J-resolved spectroscopy separates chemical shifts and coupling constants into two distinct dimensions, proving particularly valuable for analyzing complex spectra with severe peak overlap. The aforementioned experiments, along with heteronuclear single quantum coherence spectroscopy (HSQC), which maps the chemical shifts of directly detected nuclei (e.g., ^1^H) against indirectly detected nuclei (e.g., ^13^C), are commonly employed in metabolomics studies (Figure 8). In contrast, heteronuclear multiple quantum correlation spectroscopy (HMQC) and heteronuclear multiple bond correlation spectroscopy (HMBC) experiments find relatively fewer applications in this field [91,92,93,94].

### 3.3. Data Processing

To enhance the quality and interpretability of complex NMR data, preprocessing such as baseline correction, chemical shift calibration, peak alignment, and solvent peak removal is essential before the actual analysis. Next up is chemometric analysis, a traditional method that treats the signal intensity data as a multi-sample array of metabolite concentration; it is not necessary to assign the spectrum at this stage, as it is treated solely as a statistical object. A well-known problem with metabolomics data is the presence of confounding factors, particularly for blood and urine samples. Many features may be affected by age, diet, ethnicity, gender, or other factors, resulting in issues such as peak misalignment and baseline drift, which undoubtedly pose significant challenges to statistical analysis. This is why we tirelessly emphasize the importance of data preprocessing.

Multivariate statistical approaches are broadly classified into unsupervised and supervised analysis [97]. Unsupervised analysis does not rely on predefined class labels but instead uncovers potential patterns based on the inherent characteristics of the data itself. Several methods, including principal component analysis (PCA), hierarchical cluster analysis (HCA), K-means, and self-organizing maps (SOM), are available, with PCA being the simplest and most effective. PCA is a linear dimensionality reduction technique that transforms metabolomics data into a set of orthogonal (uncorrelated) principal components (PCs) by computing the eigenvalues and eigenvectors of the covariance matrix, and the first PC contains the largest part of the variance of the data set, with subsequent PC containing correspondingly smaller amounts of variance. The variance in PCs can then be visualized through the scores plot, and the specific variables that cause such variance are visualized through the loadings plot. However, the identity of the variables is unknown until further analysis is conducted. Compared to other methods mentioned above, PCA holds significant advantages in identifying potential outlier samples in metabolomics studies [45].

Nevertheless, supervised methods also play a crucial role in metabolomics research, with the aim of training models on data with clear class information (such as experimental group and control group) to identify and quantify the complex relationships between metabolites. Support vector machines (SVM), random forests (RF), linear discriminant analysis (LDA), orthogonal projections to latent structures discriminant analysis (OPLS-DA), and neural networks are commonly used supervised analyses that can effectively handle high-dimensional data [45]. Additionally, the stability of the model is evaluated, typically, by cross-validation and permutation testing, to ensure the biological relevance of putative variables (biomarker candidates). Overall, compared to unsupervised methods, supervised approaches, through precise modeling of metabolomics data, can achieve higher predictive accuracy and biological interpretability under more guided conditions.

Meanwhile, in metabolomics research, regardless of which chemometric analysis method is used, the conundrum of metabolite identification and absolute/relative quantification is unavoidable. But with the continuous updating and iteration of intelligent analytical platforms, this has gradually become simpler, even for researchers with limited expertise in NMR. In the past, numerous NMR databases have been developed to support the identification of metabolites, including the Human Metabolome Database (HMDB) [98], the Madison-Qingdao Metabolomics Consortium Database (MMCD) [99], the Biological Magnetic Resonance Data Bank (BMRB) [100], and the Magnetic Resonance Metabolomics Database (MRMD) [101]. These resources, which contain a large number of referenced NMR spectra of metabolites, also support metabolite identification through web-based submission of 1D and 2D NMR peak lists. Meanwhile, a variety of software tools have been developed to enable the automatic identification and quantification of metabolites, such as Batman, Bayesil, AQuA, MetaboMiner, ChenomX, and Bruker IVDr [102,103,104,105,106,107,108], and this field continues to grow rapidly [54,70,109].

## 4. Application of NMR-Based Metabolomics in TCM Research

As early as the 1970s, Wilson and Burlingame employed ^13^C isotope-tracer combined with ^2^H, ^1^H decoupled ^13^C-NMR to elucidate the ethanol metabolism in rats [110]. This pioneering study subsequently garnered the significant interest of other researchers. In the subsequent years, NMR was primarily applied to the detection and identification of metabolites, as well as the exploration of previously unknown metabolic processes [111,112,113]. Van der Greef, from the Netherlands Organization for Applied Scientific Research, was the first to utilize MS to study the metabolic fingerprints in urine in 1984, marking the beginning of a shift in focus toward metabolites in biological fluids [114]. Meanwhile, with the advancement of high-resolution NMR technology, scientists have attempted to apply it to the metabolic profiling analysis of various complex samples, such as serum, plasma, urine, CSF, etc., demonstrating the remarkable application potential of NMR in the analysis of complex biological fluids [115,116]. Therefore, although the concept of “metabolomics” was only formally introduced in 1999 [9], the earliest metabolomics studies can be said to have emerged in the 1980s. Over the past 20 years, NMR-based metabolomics has gained significant favor due to its non-destructive, unbiased, and highly reproducible properties.

With a history spanning thousands of years, TCM embodies the profound wisdom of the Chinese nation in combating diseases. This is exemplified by the critical role of the “Three Medicines and Three Formulations” during the COVID-19 pandemic [117]. Nevertheless, scientifically elucidating the mechanisms of TCM remains highly challenging, while NMR-based metabolomics has undoubtedly become one of the most effective approaches to overcome this dilemma. Although substantial progress has indeed been achieved (Table 1), these advancements are still insufficient and necessitate further in-depth investigation by more researchers.

### 4.1. Discovery of Syndrome/Disease Biomarkers and Evaluation of the Efficacy of TCM

The primary application of NMR-based metabolomics in TCM research is the discovery of syndrome/disease biomarkers. By comparing the metabolic profiles of healthy and diseased states, potential biomarkers can be identified, which are of great significance for early diagnosis, monitoring, and prognostic assessment.

In a recent study, Chen employed ^1^H-NMR-based metabonomics to assess the metabolic profiles associated with various TCM syndrome types of Wilson disease-related liver fibrosis, and they found that formate and acetoacetate could be observed in patients with each of these TCM syndromes [131]. Furthermore, the diagnostic values of the characteristic metabolite uridine for the phlegm and blood stasis syndrome, 3-hydroxybutyrate, pyruvate, and succinate for the liver and kidney yin deficiency syndrome, trimethylamine noxide, pyruvate, and succinate for the liver qi stagnation syndrome were confirmed. It has to be said that this study not only provides a scientific foundation for the diagnosis of TCM syndromes in Wilson disease-related liver fibrosis, but also offers unique insights for the integrated diagnosis and treatment of other diseases with TCM and Western medicine.

Tumors are categorized under the terms “stone”, “mass accumulation”, and “abscess and gangrene” in TCM, with high morbidity and mortality rates, and nasopharyngeal carcinoma (NPC) is one of the most dreaded among them. However, the most commonly used Epstein–Barr virus (EBV) serological test shows suboptimal results [173]. Zhou conducted a multicenter plasma NMR metabolomics study involving 97 NPC patients and 149 EBV-positive participants. Small, dense, very low-density lipoprotein particles (VLDL-5) and large, buoyant, low-density lipoprotein particles (LDL-1), as well as metabolic biomarkers (glucose, lactate, pyruvate, methionine, and ornithine), are found to be closely related to NPC. Based on this, an NMR-based risk prediction model was developed, which increased the positive predictive value (PPV) of NPC to 70.08% [174]. Induction chemotherapy (IC) is the primary treatment for advanced NPC, and plasma lipoprotein profiles were obtained using ^1^H-NMR before and after IC treatment. As a result, the team further found that dysregulation of plasma lipoprotein may lead to resistance to IC in NPC patients. In other words, customized lipid regulation therapy may increase the efficacy of IC, which is of great significance for the development of treatment strategies [175].

Another typical example is about depression, which is the fourth leading cause of disability globally, affecting around 20% of the world’s population [176]. Xiaoyao-San (XYS), originating in the Song dynasty, is a classic formula that disperses stagnated liver qi for relieving qi stagnation, and it has a long history of clinical use for alleviating a wide variety of depression symptoms. A recent brain metabolism study revealed that D-glutamine and D-glutamate metabolism, arginine and proline metabolism, alanine, aspartate and glutamate metabolism, taurine and hypotaurine metabolism, as well as valine, leucine, and isoleucine biosynthesis are the most influenced pathways associated with depression-like behavior. Notably, XYS significantly mediated all of these pathways except for the last one [177]. Similarly, another study integrating 16S rRNA gene sequencing and ^1^H-NMR metabolomics techniques, focusing on the gut microbiota and its metabolic profiles, elucidated that the anti-depression effects of XYS may be associated with the regulation of alpha and beta diversity of microbiota, the reduction of the abundance of harmful bacteria, i.e., the genera *Corynebacterium* and *Facklamia*, the improvement of the abundance of beneficial bacteria, especially *Lactobacillus*, and the recovery of abnormal levels of cecal metabolites (alanine, proline, lactate, and valine) [157].

### 4.2. Exploration on the Toxicity and Detoxification Mechanisms of Toxic TCM

The vague toxic components and mechanisms of TCM have led to ongoing international controversy regarding its safety, posing a huge obstacle to the development of TCM. Through NMR-based metabolomics, we can predict the occurrence of toxicity in TCM by monitoring changes in toxic markers and metabolic pathways within biological systems, elucidating the mechanisms of toxicity and detoxification.

Cinnabar, a mineral that has been used as medicine for a long time, is widely used in various prescriptions for its sedative and hypnotic effects, but at the same time, it may cause toxicity due to its high mercury content (96% as HgS) [178]. To further investigate its toxicological effects, researchers intragastrically administered cinnabar to male Wistar rats (dosed at 0.5, 2, and 5 g/kg), followed by analysis of the NMR-based metabolic profiles of blood and urine using multivariate pattern recognition techniques. Liver and kidney histopathology examinations and serum clinical chemistry analyses were also performed. Ultimately, it was revealed that cinnabar induced disturbances in energy metabolism, amino acid metabolism, and the gut microbiota environment, as well as causing mild liver and kidney damage, which may be indirectly attributed to cinnabar-induced oxidative stress [179]. Zhusha Anshen Wan (ZSASW) is an effective formula for calming the mind and soothing the nerves, composed of Cinnabar, *Coptis chinensis* French., *Angelica sinensis* (oliv.) Diels, uncooked *Rehmannia glutinosa* Libosch., and honey-fried *Glycyrrhiza uralensis* Fisch. Although cinnabar is included, clinical practice has shown that it does not exhibit the toxicity of administering equal doses of cinnabar alone, suggesting that one or more herbs in ZSASW can counteract the damage caused by cinnabar. But who exactly is responsible for this effect, and what is the mechanism behind it? To clarify this issue, an experiment was conducted based on high-resolution ^1^H-NMR, where cinnabar was combined with each of the herbs [180]. It was concluded that the metabolic profiles of the Cinnabar-*Glycyrrhiza uralensis* Fisch group and Cinnabar-*Coptis chinensis* French group were remarkably similar to the control group, while those of the Cinnabar-*Rehmannia glutinosa* Libosch group and Cinnabar-*Angelica sinensis* group were close to those of the cinnabar group. In other words, *Coptis chinensis* and *Glycyrrhiza uralensis* serve as the primary detoxification agents within ZSASW. More professionally speaking, they reverse biochemical pathways related to energy metabolism and amino acid metabolism, as well as gut microbiota disorders, thereby alleviating cinnabar-induced liver and kidney damage. This conclusion has been endorsed by other experts [133].

Realgar, a renowned TCM used to treat carbuncles, boils, insect and snake bites, intestinal parasitosis, convulsive epilepsy, and psoriasis, has a medicinal history of thousands of years. However, due to its arsenic content, a known carcinogen, it has been classified as one of the most potentially hazardous toxic TCMs. To ease public concerns, researchers have explored the toxic side effects and mechanisms of realgar [140,181]. The ^1^H-NMR metabolomic analysis of urine, serum, and liver tissue aqueous extracts highlighted the complex disruption of biochemical pathways in realgar-exposed mice, with energy metabolism, choline metabolism, and amino acid metabolism being the most closely related. These findings were further supported by clinical chemical data. Compatible detoxification is one of the unique advantages of TCM, and *Glycyrrhizae Radx et Rhizoma* is often used as an adjuvant with various toxic medicines due to its ability to regulate herbal properties and facilitate detoxification. A novel study found that the main active ingredient of *Glycyrrhizae Radx et Rhizoma*, glycyrrhetinic acid, reduces realgar-induced hepatotoxicity by dose-dependently modulating biomarkers VLDL/LDL, 3-hydroxybutyric acid, lactate, NAc, D-glucose, and choline [156].

**Figure 9:**
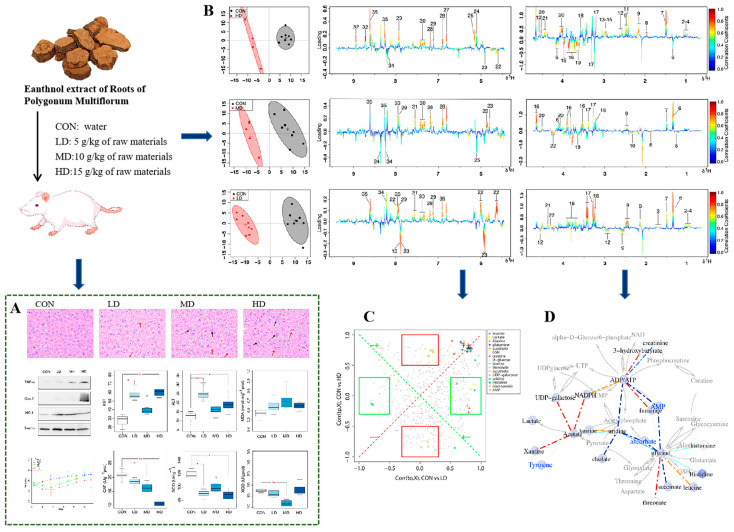
The dual nature of hepatotoxicity and hepatoprotective effects of Polygonum multiflorum (adapted from [121]). (**A**) Hepatic histopathology, biochemical parameters, and western blot results; (**B**) Analysis of ^1^H NMR spectra. Positive and negative peaks indicate a relatively decreased and increased metabolite level; (**C**) Shared and unique structures-plots. Unique features appeared near the rectangular boundaries of either the X or Y axis, while shared ones were situated diagonally; (**D**) The correlation network diagram used to decipher the biological relationships among biomarkers.

*Polygonum multiflorum* Thunb. is a popular herb that is not only considered a miracle medicine for delaying aging, but also widely used as a dietary supplement in Europe. In recent years, there have been reports that it has both hepatotoxicity and hepatoprotection, like a biological coin with two completely opposite sides [182,183]. Using ^1^H NMR metabolomics technology, Ruan investigated the mechanism of hepatotoxicity induced by *Polygonum multiflorum* Thunb., aiming to reveal the existence of a conversion between hepatotoxicity and hepatoprotection in mice during the administration of it (Figure 9). The results suggest that it induced hepatotoxicity in an apparent non-linear manner, and switches between hepatotoxicity and hepatoprotection depending on the dosage and status of the body [121]. Similar examples abound, but we will not redundantly elaborate on them here.

### 4.3. Decipher the Compatibility Rules of TCM Prescriptions

Formulas are the primary form of clinical application of TCM, and they are by no means a simple stacking of herbs but follow certain objective principles and rules, for instance, the theory of “Monarch, Minister, Assistant, and Envoy” (Jun-Chen-Zuo-Shi) and the “seven relations” (compatibility of the Seven Emotions). It can be said that these are clinical summaries formed by generations of physicians over thousands of years, struggling against nature and disease, representing the crystallization of their wisdom. The compatibility of different formulas enabling the same herbal material to exhibit distinct therapeutic effects in different contexts is traceable, but the elucidation of the underlying scientific connotations remains a major focus and challenge in the modernization of TCM [184,185]. Undoubtedly, the introduction of NMR-based metabolomics technology has provided a novel perspective and strong support for this endeavor.

In a recent study on XYS, researchers adopted the “Efficacy Compositions” research strategy, dividing the formula into two efficacy groups, i.e., the Shugan (SG) and the Jianpi (JP) groups, followed by a systematic fecal metabolomics study. The SG group consists of *Radix Bupleuri*, *Radix Paeoniae Alba*, *Radix Angelicae Sinensis*, and *Herba Menthae,* and the JP group is made up of *Rhizoma Atractylodis Macrocephalae*, *Poria*, *Rhizoma Zingiberis Recens,* and *Radix Glycyrrhizae*. As a result, 10 potential biomarkers (elevated levels of asparagine, aspartate, lactate, and propionic acid, along with decreased levels of phenylalanine, tyrosine, proline, alanine, glutamine, and glutamic acid) for depression were identified, and 8 out of 10 metabolites (phenylalanine, tyrosine, proline, asparagine, glutamate, glutamine, lactate and propionic acid) were significantly reversed by XYS, while 5 (phenylalanine, glutamate, glutamine, proline, propionic acid) and 4 biomarkers (lactate, propionic acid, glutamate and glutamine) were reversed by SG and JP, respectively. This indicates that the antidepressant effect of XYS is stronger than that of the SG and JP groups, and only the combined application of the SG and JP groups can bring about the comprehensive antidepressant effect of XYS [119].

Niuhuang Jiedu tablets (NJT), a common TCM that can be found in almost every household’s medicine drawer, are composed of Realgar, *Bovis Calculus Artificialis*, *Borneolum Synthcticum*, *Gypsum Fibrosum*, *Rhei Radix et Rhizoma*, *Scutellariae Radix*, *Platycodonis Radix,* and *Glycyrrhizae Radix et Rhizoma,* and are widely used for treating acute tonsillitis, pharyngitis, periodontitis, and mouth ulcers. It is an indisputable fact that realgar contains the toxic element arsenic, yet NJT has continued to be used with confidence, thanks to the classic TCM principle of combining different herbs to counterbalance each other and alleviate toxicity [186,187]. Similar to the research methodology of XYS, the experimental rats were divided into five groups: control, group I (treated with *Realgar*), group II (treated with *Realgar*, *Bovis Calculus Artificialis*, *Borneolum Synthcticum*, *Gypsum Fibrosum*, *Rhei Radix et Rhizoma*, *Scutellariae Radix*, *Platycodonis Radix,* and *Glycyrrhizae Radix et Rhizoma*), group III (treated with *Realgar*, *Bovis Calculus Artificialis*, *Borneolum Synthcticum,* and *Gypsum Fibrosum*), and group IV (treated with *Realgar*, *Rhei Radix et Rhizoma*, *Scutellariae Radix*, *Platycodonis Radix,* and *Glycyrrhizae Radix et Rhizoma*). Metabolomic analysis based on NMR showed that the metabolic profile of Group III was similar to those of Group I, while the metabolic profiles of Groups II and IV were close to those of the control group. The findings suggest that NJT exhibits a favorable safety profile and demonstrates potential efficacy in mitigating realgar-induced toxicity in this animal model. Specifically, *Rhei Radix et Rhizoma*, *Scutellariae Radix*, *Platycodonis Radix,* and *Glycyrrhizae Radix et Rhizoma* appear to effectively alleviate the toxicity of realgar, helping to restore normal energy metabolism, choline metabolism, amino acid metabolism, and gut microbiota balance disrupted by realgar exposure [188].

Whether discussing XYS or NJT, the purpose is to illustrate that NMR-based metabolomics aids in the interpretation of formula compatibility principles—these principles that, in turn, promote TCM to gain a larger presence on the international stage.

### 4.4. Quality Evaluation of Traditional Chinese Medicine

The quality of TCM directly determines its safety and efficacy and has become a key factor restricting the development of TCM. Professor Changxiao Liu, an academician of the CAE, proposed the concept of “quality markers” for this [189]. However, due to the vast diversity and structural complexity of secondary metabolites in plants, it is difficult to use traditional analytical methods to meet the high-throughput analysis requirements. Fortunately, NMR-based metabolomics offers a novel approach to address this issue.

Farfarae Flos has been widely used for the treatment of cough, asthma, and bronchitis. However, according to the clinical experience of TCM, only the flower buds are utilized in prescriptions, while the bloomed flowers are not. This may seem puzzling. Based on NMR-fingerprint coupled with multivariate analysis, researchers conducted a comparative study of the chemical composition and bioactivities of Farfarae Flos collected at different developmental stages. It was found that the chemical components, such as caffeic acid, sitosterone, 4,5-O-dicaffeoylquinic acid, 3,4-O-dicaffeoylquinic acid, and chlorogenic acid, were closely related to its expectorant and antitussive effects. Some primary metabolites, such as glutamic acid, fumaric acid, malic acid, acetic acid, and creatine, may work synergistically with secondary metabolites, thus influencing the biological functions of Farfarae Flos [122].

**Figure 10:**
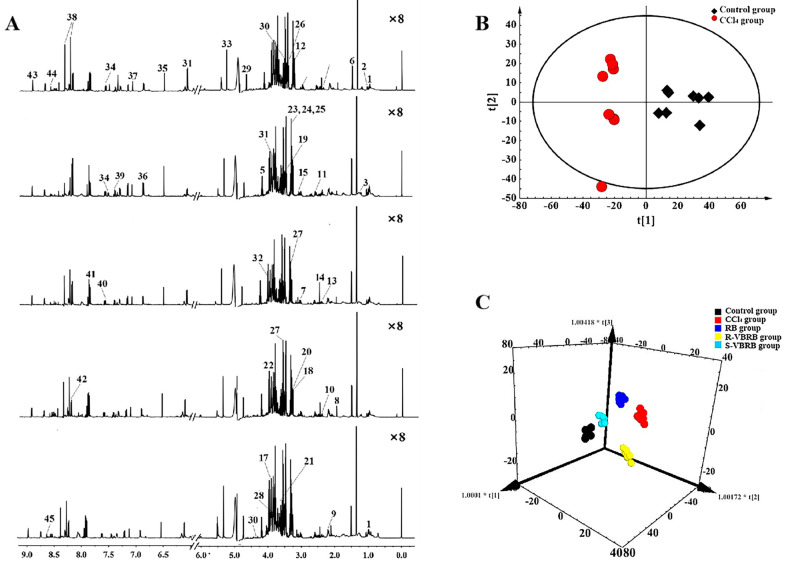
Processing RB with vinegar is necessary (adapted from [123]). (**A**) The ^1^H NMR spectra of liver extracts obtained from different groups. From top to bottom: control group, carbon tetrachloride group, RB-treated group, vinegar-baked RB by rice vinegar-treated group, and vinegar-baked RB by Shanxi vinegar-treated group; (**B**) PCA score plot based on PC1 (34.6%) and PC2 (13.5%); (**C**) The OPLS-DA score plot.

Radix Bupleuri (RB), derived from the roots of *Bupleurum chinense* DC. and *Bupleurum scorzonerifolium* Willd., is a highly sought-after herb that is commonly baked with vinegar to produce vinegar-baked RB, which is used for the treatment of liver diseases. However, traditional biochemical and pathological indicators are not sufficient enough to explain the differences in their efficacy. Therefore, Xing employed NMR-based metabolomics, a more sensitive approach, to not only confirm the necessity of processing RB with vinegar and the superiority of Shanxi vinegar over rice vinegar, but also revealed that the hepatoprotective effect is associated with the energy metabolism, lipid metabolism, ketone body metabolism, glutathione metabolism, amino acids metabolism, and nucleotide synthesis (Figure 10, Table 2) [123].

*Gastrodiae Rhizoma*, a well-known and commonly used herb, is almost as famous as ginseng and has been confirmed to alleviate chronic atrophic gastritis. The therapeutic effects of extracts from different polar parts were explored, and the results showed that the water extracts exhibited the best efficacy. In other words, the effective components of *Gastrodiae Rhizoma* are compounds of high polarity that correct disrupted energy metabolism and amino acid metabolism [190]. Regardless of the examples mentioned above, each clearly demonstrates the crucial role of NMR-based metabolomics in the quality evaluation and control of TCM.

## 5. Challenges and Prospects

As a high-precision analytical technique, the influence of NMR-based metabolomics in TCM research is still expanding. To make NMR more competitive, it is imperative to make it smaller, cheaper, and more sensitive. Placing it on the desktop, low-field and benchtop NMR seems to be feasible [191]. Obviously, its resolution and metabolite identification capabilities cannot be equivalent to high-field superconducting NMR, but the significant reduction in price and maintenance costs makes it affordable for almost every laboratory interested in it. Recently, a low-field, benchtop NMR (60 MHz) was used to characterize the alterations in the metabolic profile of fecal extracts obtained from dextran sodium sulfate-induced ulcerative colitis model mice [192]. It was found that it successfully discriminated against the model group from the healthy control group, and showed high comparability with high-field NMR (800 MHz). This undoubtedly presents a convincing solution. In addition, liquid helium is a precious non-renewable resource, and the emergence of cryogen-free superconducting magnet technology is undoubtedly an excellent solution. These systems no longer require constant liquid He or liquid N_2_ refills. Instead, it utilizes cryocoolers or closed-cycle helium refrigerators to bring magnet temperatures close to 4 K, without worrying about a decline in its identical performance [193,194]. Of course, if cost and size are not an issue, an ultra-high-field magnet is an excellent development direction for the future. Currently, 1.2 GHz NMR instruments have been put into use, and it is said that they can significantly improve the S/N, which is crucial for concentration-limited biological samples [195]. The development of the microcoil probe is also a promising avenue to improve the sensitivity of NMR in biofluids in the future [196], and both the reported 1.5 mm HTS NMR probe and the 1 mm HRμMAS probe have doubled our confidence in this path [197,198,199]. It must be mentioned that hyperpolarization offers an approach for enhanced sensitivity with even higher potential [200].

The multi-component and multi-target characteristics of TCM endow it with immense potential, yet simultaneously present significant challenges, a fact that is widely acknowledged. Another critical bottleneck is the difficulty in obtaining high-quality NMR spectra from complex mixtures such as biological fluids and herbal materials, as well as the considerable challenges in data interpretation, particularly for researchers who are not specialized in physics but are interested in NMR-based metabolomics. Fortunately, amid the wave of the artificial intelligence (AI)-led Fourth Industrial Revolution, the integration of AI with NMR-based metabolomics has brought unprecedented opportunities for the research of TCM. For instance, AI-driven algorithms enable automated and real-time parameter optimization, thereby minimizing experimental variability and improving reproducibility. At the level of data analysis, we can introduce deep learning algorithms, such as Convolutional Neural Network (CNN) and Long Short-Term Memory Network (LSTM), to process high-dimensional and complex metabolomic data with greater precision, facilitating rapid metabolite annotation. AI also serves as a key enabler in the burgeoning field of multi-omics integration research. By leveraging AI to analyze multi-omics data synergistically, we can effortlessly unveil the panoramic interaction networks underlying the effects of TCM, offering profound insights into its intricate molecular regulatory mechanisms with unprecedented clarity. In summary, with the continuous advancements of big data, machine learning, and AI algorithms, the methods of data acquisition and analysis in NMR-based metabolomics have undergone revolutionary changes. This interdisciplinary integration is expected to significantly accelerate the construction of the bridge between TCM and modern science. At present, the Bruker Avance IVDr metabolic analysis platform is the most representative example. According to reports, it enables high-throughput, fully standardized, and automated workflows, allowing researchers to obtain reliable qualitative and quantitative results without the need to analyze tedious and difficult spectra, and it is considered a reliable tool for targeted and untargeted metabolomic study of human body fluids (plasma, serum, urine, and cerebrospinal fluid) [152,155]. If more high-performing software or platforms like it can be developed in the future, research in metabolomics of TCM will thrive even more. Macroscopically speaking, integrating AI into the realm of TCM and endowing its ‘traditional paradigms’ with modern digital ‘wings’ will undoubtedly infuse new vitality into the ancient TCM wisdom [201,202,203].

Last but not least, the current healthcare system remains predominantly disease-centered, structured around treatment modalities designed for population-wide effectiveness. This “one-size-fits-all” paradigm fundamentally contradicts the traditional philosophy of syndrome-differentiated therapy. The application of NMR-based metabolomics for accurate biomarker identification in various syndromes/diseases, when integrated with Western diagnostic modalities (e.g., advanced imaging), enables precise stratification of disease states and progression patterns. This approach may ultimately facilitate personalized therapeutic regimens of prescription, representing a transformative advancement in healthcare. However, this precision medicine model with Chinese characteristics remains in its infancy. Most NMR-based metabolomics studies of TCM are currently confined to laboratory settings, and their clinical translation still presents substantial challenges.

## 6. Conclusions

NMR-based metabolomics has undoubtedly emerged as one of the most robust and promising tools for scientifically elucidating the theoretical system of TCM, owing to its distinctive advantages, including multi-nuclear detection capability, non-destructive analytical properties, absolute quantification potential, and exceptional structural elucidation capacity. The ongoing efforts dedicated to improving NMR hardware and software are expected to make NMR-based metabolomics even more engaging and productive in the study of TCM. Moreover, AI is revolutionizing the research paradigm of TCM, and its integration with NMR-based metabolomics is poised to serve as a pivotal bridge between traditional and modern medicine, facilitating the ‘digital-intelligent’ transformation of TCM.

Currently, NMR-based metabolomics has shown initial success in areas such as the study of syndrome/disease biomarkers, the interpretation of formula compatibility principles, and the quality evaluation of TCM. In the future medical system, precision medicine is destined to play a crucial role. The syndrome differentiation and treatment of TCM represent a dynamic and individualized diagnostic and therapeutic process, embodying the foundational concepts of precision medicine and showcasing distinct characteristics and advantages. Against the backdrop of the global emphasis on TCM, we must seize the opportunity, focus on clinical needs, respect the principles of TCM, integrate multidisciplinary knowledge, and establish a precision diagnosis and efficacy evaluation model for TCM. This will help bridge the gap between traditional and modern medicine. In the flourishing era of omics technologies, we should fully utilize NMR-based metabolomics to precisely differentiate syndromes, accurately understand formulas, and evaluate their efficacy, thereby constructing a precision medicine model with Chinese characteristics. However, the vast majority of current research is conducted in laboratories, and there is still a considerable distance to cover in translating these experimental findings into clinical applications. Nevertheless, as long as we work together, the future is undoubtedly promising.

## Figures and Tables

**Table 1:** Classic cases of NMR-based metabolomics research of TCM in recent years.

Magnetic Field Strength (MHz)	Technique (s)	Sample	TCM(s) Involved	Research Objective	Reference
600	^1^H, COSY, HSQC	Danshen injection	Danshen	Taking Danshen Injection as a case, establish a rapid and comprehensive method for evaluating the quality of Traditional Chinese Medicine injections.	[118]
600	^1^H	fecal	Xiaoyao-San	Investigate the compatibility rule of the antidepressant effect of Xiao-Yao-San by using both the “Efficacy Compositions” research strategy and fecal metabolomics approach.	[119]
400	^1^H	Extract of Ginseng	ginseng	Metabolomic quality control of commercial Asian ginseng and cultivated and wild American ginseng using ^1^H NMR and multi-step PCA.	[120]
500	^1^H	serum	*Polygonum multiflorum* Thunb.	Study the mechanism of *Polygonum multiflorum* Thunb. induced hepatotoxicity and confirm the existence of a conversion between hepatotoxicity and hepatoprotection during the administration.	[121]
600	^1^H	serum	Farfarae Flos	Comparison of the antitussive and expectorant effect of Farfarae Flos collected at different stages.	[122]
600	^1^H	liver	Radix Bupleuri.	Compare the hepatoprotective efficacy of raw and vinegar-baked Radix Bupleuri.	[123]
600	^1^H, HSQC, COSY	urine	HuangqiJianzhong-Tang	Research the mechanism of HuangqiJianzhong-Tang in combating chronic atrophic gastritis.	[124]
—	^1^H	brain	Naozhenning granule	Reveal the protective effect and the potential active components of Naozhenning granule against traumatic brain injury.	[125]
600	^1^H, COSY	urine	*Rubus suavissimus* S. Lee	Study the antidiabetic effects of *Rubus suavissimus* S. Lee in streptozotocin-induced type 1 diabetes mellitus rats.	[126]
600	^1^H, HSQC, TOCSY	serum	HuangQi, DanShen	Explore the active mechanism of HuangQi-DanShen against cerebral ischemia.	[127]
600	^1^H	serum, urine	Epimedii Folium	Investigate the efficacy and potential mechanism of icariin, the main prenylflavonoid of Epimedii Folium, in ameliorating chronic kidney disease.	[128]
500	^1^H	plasma, urine	Moutan Cortex charcoal	Explore the protective effect of Moutan Cortex charcoal on blood-heat and hemorrhage in rats.	[129]
—	^1^H	CSF	BuYangHuanWu Decoction	Multi-technology integration to reveal the effects of BuYangHuanWu Decoction on neurodegenerative diseases.	[130]
500	^1^H, HSQC, COSY	plasma, urine, brain	Naodesheng	Determine the protective Effect of a combination of multiple components derived from Naodesheng on ischemic stroke in rats.	[95]
600	^1^H	serum	—	Evaluate the metabolic profiles associated with different TCM syndromes of liver fibrosis related to Wilson’s disease and analyze the diagnostic and predictive capabilities of various metabolites.	[131]
600	^1^H	liver, spleen, kidneys	Saffron essential oil	Study the effect and related molecular mechanisms of Saffron essential oil against depression.	[132]
600	^1^H	serum, urine	*Coptis chinensis* Franch	Explore the protective effects of *Coptis chinensis* Franch and its main component, berberine, on cinnabar-induced hepatotoxicity and nephrotoxicity.	[133]
500	^1^H, HSQC, TOCSY	plasma, urine	Baoyuan decoction	Delineates the therapeutic effects of Baoyuan decoction on isoproterenol-induced cardiac hypertrophy.	[96]
600	^1^H	urine	Guilingji	Investigate the protective effects of Guilingji on the testicular dysfunction of aging rats, as well as its regulating effects on metabolic disorders in natural aging rats.	[134]
600	^1^H, COSY, TOCSY, HMBC, JRES NMR	liver, kidney	Qijian mixture	Assess the safety and efficacy of Qijian mixture in the treatment of type 2 diabetes by metabonomics, gut microbiota, and system pharmacology.	[135]
500	^1^H	urine	You-Gui Pill	Uncover the targets and metabolic pathways of the You-Gui Pill in Treating Kidney-Yang deficiency syndrome.	[136]
—	^1^H	liver	Rhizoma Paridis saponins	Study the potential mechanism of Rhizoma Paridis saponins-induced hepatotoxicity in rats.	[137]
500	^1^H	urine, serum	kernels of castor beans	Study the chronic toxicity of crude ricin from kernels of castor beans on rats.	[138]
600	^1^H	urine	*Rhizoma glycyrrhizae*, *Aconitum carmichaelii* Debx.	Explain how *Rhizoma glycyrrhizae* alleviates the toxicity of *Aconitum carmichaelii* Debx.	[139]
600	^1^H	plasma, urine	realgar	Elucidate the mechanism of sub-chronic hepatotoxicity induced by realgar	[140]
600	^1^H	urine, serum	Niuhuang Jiedu Tablet	Analyze the metabolic profiling of the acute toxicological effects of the realgar (As_2_S_2_) combined with other herbs in Niuhuang Jiedu Tablet.	[141]
600	^1^H, COSY, TOCSY, HSQC, HMBC, JRES NMR	serum	Xue-sai-tong injection	Integrate candidate metabolites and biochemical factors to elucidate the action mechanism of Xue-sai-tong injection.	[142]
500	^1^H	brain	Huang-Lian-Jie-Du-Decoction	Elucidate the components of Huang-Lian-Jie-Du-Decoction that act synergistically to exert protective effects in a rat ischemic stroke model.	[143]
600	^1^H, COSY, TOCSY, HSQC, HMBC, JRES NMR	plasma, urine	—	Discover the potential mechanism of dioscin against hyperuricemia in mice.	[144]
600	^1^H	liver, small intestine	Xiaobugan decoction	Explore the prophylactic and hepatoprotective effects of Xiaobugan decoction and explore its related molecular mechanisms.	[145]
600	^1^H	liver	Xiaoyao-San	Elucidate the potential link between the antidepressant and hepatoprotective effects of Xiaoyao-San.	[146]
600	^1^H	urine	—	Investigate the specific changes in metabolites and proteins of Kidney-Yin Deficiency Syndrome patients with diabetes mellitus (DM) in China.	[147]
500	^1^H	serum, urine, brain	*Epimedium brevicornum* Maxim.	Study the antidepressant-like effect and the possible mechanisms of icariin in a rat model of corticosterone-induced depression.	[148]
500	^1^H	urine	*Coptis chinensis* Franch.	Combination of ^1^H NMR and GC-MS-based metabonomics to study the toxicity of *Coptidis rhizome* in rats.	[149]
500	^1^H	urine, serum	*Arisaematis rhizoma*	Toxicity assessment of *Arisaematis rhizoma* in rats by an NMR-based metabolomics approach.	[150]
500, 600	^1^H	serum, plasma	—	Brings new insights into human metabolic biology by using high-throughput metabolomics platforms for Genome-wide association analyses.	[151]
600	^1^H	serum, plasma	—	Integrative Modeling of Plasma Metabolic and Lipoprotein Biomarkers of SARS-CoV-2 Infection in Spanish and Australian COVID-19 Patient Cohorts.	[152]
600	^1^H	plasma	—	Confirm the feasibility of fecal microbiota transplantation plus anti-PD-1 immunotherapy in advanced melanoma.	[153]
600	^1^H	urine, plasma	*Xanthium strumarium* L.	Revealing the hepatotoxic constituents and toxicological mechanism of *Xanthium strumarium* L. fruits.	[154]
600	^1^H, JRES NMR	plasma	aronia	Investigated if aronia juice tolerability was associated with changes in intestinal microbiota and metabolites.	[155]
600	^1^H	plasma	realgar	Explore the hepatoprotective effects of glycyrrhetinic acid on realgar-induced sub-chronic hepatotoxicity in mice.	[156]
600	^1^H	cecal samples	Xiaoyao-san	Study the underlying mechanism of the anti-depressant effects of Xiaoyao san from the perspective of cecal microbiota and metabolites.	[157]
500	^1^H	liver, kidney, serum	Ershiwuwei Shanhu Pill	Investigate the safety profile of Ershiwuwei Shanhu Pill, a classic Tibetan medicinal formulation.	[158]
500	^1^H	kidney, serum, testicle	Wu-Zi-Yan-Zong-Wan	Elucidate the potential biomarkers and metabolic pathways involved in the treatment of oligozoospermia with Wu-Zi-Yan-Zong-Wan, providing a basis and guidance for its clinical application.	[159]
600	^1^H, HSQC, TOCSY	skin tissues	Huiyang Shengji formula	Provide new insights into the mechanisms of the healing effects of the Huiyang Shengji formula in the treatment of diabetic skin ulcer.	[160]
600	^1^H, HSQC, COSY	urine	Huangqi Jianzhong Tang	Integrate urine metabolomics and SystemsDock to screen out the material basis of Huangqi Jianzhong Tang against chronic atrophic gastritis.	[161]
600	^1^H	serum	Ba-Wei-Long-Zuan Granule	Reveal the active ingredients and anti-arthritic Mechanisms of Ba-Wei-Long-Zuan Granule.	[162]
600	^1^H	serum	San-Huang-Xie-Xin decoction	Combine ^1^H-NMR metabolomics and biochemical assays to investigate the anti-stress effects and underlying mechanisms of San-Huang-Xie-Xin decoction on restraint-stressed mice.	[163]
600	^1^H	urine, plasma, spleen	Sijunzi decoction	Utilize NMR- and MS-based metabolomics technologies to investigate the preventive effect of Sijunzi decoction on mitomycin C-induced immunotoxicity.	[164]
850	^1^H	striatum	Bushen Huoxue Huazhuo Recipe	Analyze the effects of two different interventions, penicillamine and Bushen Huoxue Huazhuo Recipe, on metabolites in striatal tissues of Wilson’s disease copper-loaded rats, providing valuable references for future integrated Western and Chinese medicine interventions targeting Wilson’s disease-related nerve injury.	[165]
600	^1^H	serum	Astragali Radix	Elucidate the potential material basis and mechanism of Astragali Radix against nephrotic syndrome.	[166]
600	^1^H	urine, serum	*Dimocarpus longan* Lour.	Reveal the therapeutic effect of the leaves of *Dimocarpus longan* Lour. on type 2 diabetes from a metabolomic perspective.	[167]
700	HSQC	*Scutellaria* crude extracts	*Scutellaria*	Identify anti-non-small cell lung cancer bioactive compounds in *Scutellaria* via NMR-Based Metabolomic analysis of pharmacologically classified crude extracts.	[168]
—	^1^H	liver	Yinchen Sini decoction	Reveal the molecular mechanism of Yinchen Sini decoction in the treatment of acute liver injury using integrated network analysis and metabolomics.	[169]
600	^1^H, COSY	feces	Danggui Sini decoction	Integrate NMR metabolomics and 16S rRNA gene sequencing techniques to clarify the intervention of Danggui Sini decoction on collagen-induced rheumatoid arthritis.	[170]
600	^1^H	kidney	Xiaoyao-san	Reveal the compatibility of Xiaoyaosan from the perspective of the “gut-liver-kidney” axis based on the strategy of the “Efficacy Group”.	[15]
600	^1^H	serum	Gushudan	Explore the preventive mechanism of Gushudan on kidney-yang-deficiency-syndrome through an integrated approach combining pharmacodynamics, 1H NMR serum metabolomics, and endogenous network pharmacology analysis.	[171]
600	^1^H	feces	Gushudan	Combine 1H NMR fecal metabolomics and 16S rRNA gene sequencing to reveal the protective effects of Gushudan on kidney-yang-deficiency-syndrome rats via the gut–kidney axis.	[172]

**Table 2:** Assignments of ^1^H NMR spectra peaks (adapted from [123]).

No.	Metabolites	Chemical Shift (ppm)
1	isoleucine	0.95 (t), 1.01 (d), 1.99 (m)
2	leucine	0.96 (d), 0.97 (d)
3	valine	0.99 (d), 1.04 (d)
4	*β*-hydroxybutyrate	1.20 (d), 2.41 (d), 2.31 (d)
5	lactic acid	1.33 (d), 4.12 (q)
6	alanine	1.48 (d)
7	ornithine	1.73 (m), 1.93 (m), 3.05 (t), 3.77 (t)
8	acetic acid	1.92 (s)
9	glutamic acid	2.05 (m), 2.34 (m), 3.75 (m)
10	glutamine	2.14 (m), 2.44 (m), 3.77 (m)
11	methionine	2.14 (s), 2.14 (m), 2.64 (t), 3.85 (m)
12	glutathione disulfide	2.17 (m), 2.54 (m), 2.95 (m), 3.25 (m), 2.98 (dd), 3.32 (dd)
13	pyruvic acid	2.37 (s)
14	succinic acid	2.41 (s)
15	aspartic acid	2.70 (dd), 2.82 (dd), 3.90 (dd)
16	trimethylamine	2.88 (s)
17	creatine	3.04 (s), 3.93 (s)
18	choline	3.21 (s)
19	*scyllo*-inositol	3.36 (s)
20	phosphatidylcholine	3.22 (s)
21	glycerophosophocholine	3.23 (s), 3.63 (m), 4.30 (m)
22	phosphoethanolamine	3.23 (t), 3.99 (m)
23	trimethylamine N-oxide	3.27 (s)
24	taurine	3.27 (t), 3.43 (t)
25	betaine	3.27 (s), 3.90 (s)
26	methanol	3.36 (s)
27	glycine	3.56 (s)
28	oxidized glutathione	3.78 (t), 2.17 (m), 2.54 (m), 2.98 (dd)
29	*β*-glucose	3.90 (dd), 4.65 (d)
30	adenosine	4.28 (q),4.45 (t),6.10 (d),8.24 (s), 8.35 (s)
31	inosine	4.28 (q), 4.45 (t), 6.10 (d)
32	mannose	5.19 (d), 3.94 (m)
33	α-glucose	5.23 (d)
34	uracil	5.8 (d), 7.55 (d)
35	fumaric acid	6.53 (s)
36	tyrosine	6.91 (d), 7.20 (d)
37	histidine	7.11 (s), 7.90 (s)
38	phenylalanine	7.32 (m), 7.42 (m)
39	uracil	7.55 (d)
40	nicotinurate	7.60 (dd), 8.72 (d), 8.94 (s)
41	uridine	7.88 (d), 5.92 (d), 4.36 (m), 4.24 (t)
42	hypoxanthine	8.20 (s), 8.22 (s)
43	nicotinic acid	8.24 (d), 8.72 (d), 8.94 (s)
44	inosine monophosphate	8.58 (s)
45	adenosine monophosphate	8.61 (s), 8.27 (s), 6.15 (d)

## Data Availability

Not applicable.

## Abbreviations

The following abbreviations are used in this manuscript:

TCM	Nuclear magnetic resonance
NMR	Mass spectrometry
MS	Radio frequency
RF	Cerebrospinal fluid
CSF	Carr-Purcell-Meiboom-Gill
CPMG	Signal-to-noise ratio
S/N	Nuclear Overhauser enhancement spectroscopy
NOESY	1H-1H correlation spectroscopy
COSY	1H-1H total correlation spectroscopy
TOCSY	Heteronuclear single quantum coherence spectroscopy
HSQC	Heteronuclear multiple quantum correlation spectroscopy
HMQC	Heteronuclear multiple bond correction spectroscopy
HMBC	Principal component analysis
PCA	Nasopharyngeal carcinoma
NPC	Induction chemotherapy
IC	Xiaoyao-San
XYS	Zhusha Anshen Wan
ZSASW	The Shugan group
SG	The Jianpi group
JP	Niuhuang Jiedu tablet
NJT	Radix Bupleuri
RB	Artificial intelligence
AI	Nuclear magnetic resonance
